# Interaction between HIV and its host: role for viral and cellular sncRNA

**DOI:** 10.1186/1742-4690-8-S2-O12

**Published:** 2011-10-03

**Authors:** Monsef Benkirane

**Affiliations:** 1 Laboratoire de Virologie Moléculaire, Institut de Génétique Humaine, 34296 Montpellier, France

## 

The primate lentivirus auxiliary protein Vpx counteracts an unknown restriction factor that renders human dendritic and myeloid cells largely refractory to HIV-1 infection. We recently identified Samhd1 as this restriction factor. Samhd1 is a protein involved in Aicardi-Goutière Syndrome (AGS), a genetic encephalopathy with symptoms mimicking congenital viral infection that has been proposed to act as a negative regulator of the ínterferon-stimulated DNA response. We show that Vpx induces proteasomal degradation of Samhd1. Silencing of Samhd1 in non-permissive cell lines alleviates HIV-1 restriction and is associated with a significant accumulation of viral DNA in infected cells. Concurrently, overexpression of Samhd1 in sensitive cells inhibits HIV-1 infection. The putative phosphohydrolase activity of Samhd1 is likely required for HIV-1 restriction. Vpx-mediated relief of restriction is abolished in Samhd1 negative cells. Finally, silencing of Samhd1 dramatically increases susceptibility of MDDCs to infection. Altogether, these results demonstrate that Samhd1 is an anti-retroviral protein expressed in cells of the myeloid lineage and inhibiting an early step of the viral life cycle.

